# Atherosclerotic and thrombotic genetic and environmental determinants in Egyptian coronary artery disease patients: a pilot study

**DOI:** 10.1186/s12872-016-0456-3

**Published:** 2017-01-13

**Authors:** Manal S. Fawzy, Eman A. Toraih, Nagwa M. Aly, Abeer Fakhr-Eldeen, Dahlia I. Badran, Mohammad H. Hussein

**Affiliations:** 1 Department of Medical Biochemistry, Faculty of Medicine, Suez Canal University, Ismailia, Egypt; 2 Department of Histology and Cell Biology (Genetics Unit), Faculty of Medicine, Suez Canal University, Ismailia, Egypt; 3 Clinical Pathology Department, Faculty of Medicine, Sohag University, Sohag, Egypt; 4 Pulmonologist, Ministry of Health, Cairo, Egypt

**Keywords:** Coronary artery disease, Gene variants, Gene-disease interactions, Polymorphism

## Abstract

**Background:**

Coronary artery disease (CAD) is the leading cause of morbidity and mortality worldwide. Multiple genetic variants in combination with various environmental risk factors have been implicated. This study aimed to investigate the association of twelve thrombotic and atherosclerotic gene variants in combination with other environmental risk factors with CAD risk in a preliminary sample of Egyptian CAD patients.

**Methods:**

Twenty three consecutive CAD patients undergoing diagnostic coronary angiography and 34 unrelated controls, have been enrolled in the study. Genotyping was based on polymerase chain reaction and reverse multiplex hybridization. Five genetic association models were tested. Data distribution and variance homogeneity have been checked by Shapiro-Wilk test and Levene test, respectively; then the appropriate comparison test was applied. Spearman’s rank correlation coefficient was used for correlation analysis and logistic regression has been performed to adjust for significant risk factors. Clustering the study participants according to gene-gene and gene-environment interaction has been done by Detrended Correspondence Analysis (DCA).

**Results:**

The univariate analysis indicated that the five variants; rs1800595 (*FVR2;* factor 5), rs1801133 (*MTHFR*; 5,10-methylenetetrahydrofolate reductase), rs5918 (*HPA-1;* human platelet antigen 1), rs1799752 (*ACE;* angiotensin-converting enzyme), and rs7412 and rs429358 (*ApoE;* apolipoprotein E) were significantly associated with CAD susceptibility under different genetic models. Multivariate analysis revealed clustering of the study population into three patient groups (P) and one control group. *FVR*2 was the most variant associated with CAD patients, combined with the factor V Leiden (*FVL*) variant in P1 cluster and with both *ACE* and *MTHFR* 667C > T in P2. Whereas, P3 was mostly affected by both *MTHFR* 667C > T and *FXIII* (factor 13) V89L mutations. When combined with traditional risk factors, P1 was mostly affected by dyslipidemia, smoking and hypertension, while P2 was mostly affected by their fasting blood sugar levels and *ApoE* variant.

**Conclusions:**

Taken together, these preliminary results could have predictive value to be applied in refining a risk profile for our CAD patients, in order to implement early preventive interventions including specific antithrombotic therapy. Further large scale and follow-up studies are highly recommended to confirm the study findings.

**Electronic supplementary material:**

The online version of this article (doi:10.1186/s12872-016-0456-3) contains supplementary material, which is available to authorized users.

## Background

Coronary artery disease (CAD) is the leading cause of morbidity and mortality worldwide [1]. In Egypt it is responsible for 21% of deaths [2] and reported a prevalence of 8.3% [3]. It is one of the complex diseases that are multifactorial or polygenic disorders, caused by multiple genetic variants with low penetrance in combination with various environmental and lifestyle factors [4]. CAD is characterized by long-term atheromatous plaque formation which culminates into atherothrombotic obstructive lesions leading to tissue damage [5]. Atherosclerosis and thrombosis are the two manifestations underlying CAD. It is known that events such as endothelial dysfunction, inflammation, abnormal lipoprotein and homocysteine metabolism, as well as dysfunctional coagulation and fibrinolysis play a key role in the pathogenesis of CAD [6]. In recent years, several genetic variants robustly associated with CAD have been detected by genome-wide association studies (GWAS), mainly in people of European descent [7]. In order to unravel the genetic profile of a sample of the Egyptian CAD patients with respect to these susceptibility loci, twelve variants were analyzed; Additional file 1: Table S1.

Seven polymorphisms associated with thrombotic risk; (i) Factor V (*FV*): FV Leiden G1691A (R534Q), leads to activated protein C (APC) resistance on the cleavage of factor V and occurs in 20–50% of patients with venous thromboembolism (VTE). It represents one of the most important genetic risk factors for inherited thrombophilia that increases in homozygous state. The contribution of this polymorphism to the pathogenesis of CAD remains controversial [8, 9] (ii) *FVR2* haplotype A4070G (H1299R) was found to be in complete linkage disequilibrium with Factor V Leiden and may confer mild APC resistance. When interacting with the Factor V Leiden mutation could produce a more severe APC resistance phenotype and increased the risk for VTE associated with Factor V Leiden by approximately 3-fold [10]. (iii) Prothrombin, (*PTH*; Factor II) G20210A; the A allele was previously shown to be associated with increased prothrombin levels due to an alteration in the mRNA processing. Its presence with a hypercoagulable state, imply a moderate association with a higher CAD risk [11, 12]. In addition, A allele carriers had about 3-fold elevated risk for cerebral and deep vein thrombosis. This risk increased significantly when combined with FV Leiden [13]. (iv) Factor XIII (*FXIII*) 163G > T (V34L); most of clinical studies have demonstrated a lower incidence of both venous and arterial thrombosis in carriers of the mutant allele (34Leu) compared to Val/Val carriers [14]. This could be explained by lower plasma level of FXIII in carriers of L34 with increased its activation that result in less stabilized and thinner fibrin clot due to easy dissociation of its 2A and 2B subunits [15] (v) 5,10-Methylenetetrahydrofolate Reductase (*MTHFR*) C677T and (vi) *MTHFR* A1298C. The 677TT homozygosity and 677CT/1298 AC compound heterozygote genotypes have been shown to have decreased *MTHFR* activity and higher predisposition to arterial and venous thrombosis in the presence of additional risk factors [16, 17]. (vii) Plasminogen Activator Inhibitor 1 (*PAI-1*, Serpin E1) 4G/5G; the 4G allele was previously found to be associated with higher *PAI-1* transcription rates and considered to be a mild risk factor for VTE and myocardial infarction (MI) [18].

Other five variants associated with atherosclerosis; (viii) Beta-Fibrinogen (*FGB*) -455G > A; confers elevated β-fibrinogen plasma levels, which are directly related to increase the risk of acute coronary syndromes (ACS) [19], premature MI [20] and ischemic stroke [21]. However controversial findings of some researchers that show a protective role of this polymorphism against non-fatal acute MI [22] still present. (ix) Human Platelet Antigen 1 (*HPA-1*; Gp IIIa; integrin b3) L33P (1a/b); studies have shown that, in the normal population, the *HPA-1*b phenotype results in increased platelet aggregation and fibrinogen binding, thus enhancing the risk of MI [23, 24]. (x) Angiotensin-Converting Enzyme (*ACE*) 287 bp insertion/deletion (I/D): D allele has been associated with the elevated *ACE* activity and plasma levels; thereby represents a risk factor for MI in older patients and in smokers [25].)xi**)** Apolipoprotein B (*Apo B*) R3500Q, that is considered one of the most common single site mutations in the human *ApoB* gene; results in mild to severe hypercholesterolaemia and an increased risk for early onset atherosclerosis [26]. (xii) Apolipoprotein E (*Apo E*) E2/E3/E4; an important predictor of the plasma lipid profile with E2 shows the lowest and E4 shows the highest LDL (low density lipoprotein) and total cholesterol levels; E4 allele was associated with increased susceptibility to early-onset MI, particularly in smokers [27].

Taken together, this study aimed to assess the presence of previously mentioned mutations and polymorphisms in a sample of Egyptian CAD patients and to correlate the co-existence and combination of these variants with the clinical and laboratory risk factors in attempt to be helpful in refining a risk profile for our CAD patients, in order to determine the need for and the intensity of follow-up or to influence decisions about staged implementation of preventive interventions with behavioral or drug therapy.

## Methods

### Study participants

The current observational case-control study included consecutive 23 unrelated Egyptian stable CAD patients undergoing diagnostic coronary angiography and 34 controls. Patients were recruited from the Cardiology Department, Suez Canal University (SCU) Hospital, Ismailia, Egypt, during the period between October 2015 and February 2016. Diagnosis of CAD was based on detailed history taking via a structured interview, thorough clinical examination, resting electrocardiography and echocardiography, followed by coronary angiography. Patients with congenital heart disease or non-atherosclerotic coronary artery disease were excluded. Unrelated healthy blood donors were included as controls. They had no past history of cardiovascular problems and had a normal resting ECG. They were included irrespective of concomitant risk factors for CAD such as obesity, family history of CAD, hypertension, diabetes mellitus, etc. Selective coronary angiography was not performed for controls following our institutional ethical guidelines that do not permit to apply invasive procedures for controls in research work. The study was conducted in accordance with the guidelines in the Declaration of Helsinki and approved by the Medical Research Ethics Committee of SCU (approval no. 2734). Written informed consent was obtained from all participants.

### Cardiovascular disease (CVD) risk assessment

Cardiovascular risk factors were recorded as described previously [28]. Hypertension was defined if the patient was receiving anti-hypertensive drugs or blood pressure exceeded 140/90 mmHg in repeated measurements. Diabetes was considered as a blood sugar ≥ 200 mg/dl on admission, fasting glycemia ≥ 126 mg/dl in two determinants or prior prescription of hypoglycemic drugs. Dyslipidemia was identified if had at least one of the following; triglycerides (TG) ≥ 150 mg/dl, total cholesterol (TC) >200 mg/dl, high density lipoprotein (HDL) <40 mg/dl in males and <50 mg/dl in females or receiving lipid-lowering drugs. Height and weight were measured in all participants and the body mass index (BMI) was calculated. BMI >25 and >30 were considered overweight and obesity, respectively. Individuals who smoked regularly during the previous 12 months were classified as smokers. Other risk factors as lack of exercise and family history of premature CAD (first degree male relatives <55 years or females <65 years) were documented [29].

Multiple methods were used for risk assessment: (1) A qualitative individual risk factor approach in which the sum of risk factors was counted using risk factor categories [30]. (2) A quantitative estimate; “Global CAD risk model” using gender-specific coronary disease prediction algorithm which estimates multivariable CAD risk based on the magnitude or degree of the risk factors [31]. The researchers tried to follow the GRIPS (genetic risk prediction studies) guidelines in reporting the current studied data [32], although our study is not a predictive one, but is considered as a preliminary step in risk assessment in our CAD population.

### Echocardiography

A two-dimensional conventional echocardiographic study was performed on all patients using the standard views to exclude the presence of structural heart disease using a commercially available system (General Electric Healthcare Company, Vivid seven Dimensions Vingmed and Horten- Norway) with a 2.5-MHz phased array probe. All echocardiographic data [i.e. the systolic (ejection fraction) and diastolic (grade) functions] were interpreted by two independent experts in echocardiography.

### Coronary angiography

Selective coronary cineangiography was performed for all patients using standard, modified Seldinger’s technique [33, 34]. Significant obstructive lesions were diagnosed by visual estimation as the presence of ≥ 50% luminal diameter stenosis in at least one major epicardial coronary artery; left main coronary artery (LMCA), left anterior descending (LAD) coronary artery, right coronary artery (RCA), and circumflex coronary artery (Cx) [35]. Basal angiographic characteristics of patients, such as the location and number of diseased vessels were documented [36]. Multivessel disease (MVD) was defined if ≥ 50% luminal narrowing extended to involve two or three major coronary arteries. Vessel scores ranged from 0 to 3, depending on the number of involved vessels.

A modified Gensini score; a quantitative scoring system applied in our hospital protocols, was used to assess CAD extent and severity from coronary angiograms, taking into account the vessel affected and lesion location, the cumulative effect of multiple lesions, the degree of stenosis, and the influence of collaterals [34, 37]. Each item was assigned a number of points. The score for each vessel was calculated separately, and eventually all are added up to give the total score. The following equation was used: severity score x the segment location multiplying factor x collateral adjustment factor [37]. Scores were determined and interpreted by two independent angiographers who were blinded to the clinical data. A Gensini score value greater than 20 was defined as a high Gensini score [38].

### Specimen collection and the laboratory investigations

Five milliliter overnight fasting blood samples were collected on both trisodium EDTA (1 mg/ml) tubes for subsequent genetic analysis study and Serum Separator Vacutainer Tubes II (Becton Dickinson Plymouth). After blood clotting, the latter tubes were centrifuged immediately at 2500 rpm for 20 min and the separated serum were aliquoted into eppendorfs (1 ml per aliquot) and stored at -20 °C for subsequent biochemical assay. Fasting blood glucose (FBG), serum total cholesterol (TC), high density lipoprotein cholesterol (HDL-c) and serum triglycerides (TG) were determined by the enzymatic method using Hitachi 912 automated chemistry analyzer (Roche Diagnostics Co, Mannheim, Germany). Low density lipoprotein-cholesterol (LDL-c) value was calculated by the Friedewald’s equation [39] as all the study serum TG samples were < 400 mg/dl.

### DNA extraction and In vitro amplification

**Fig. 1:**
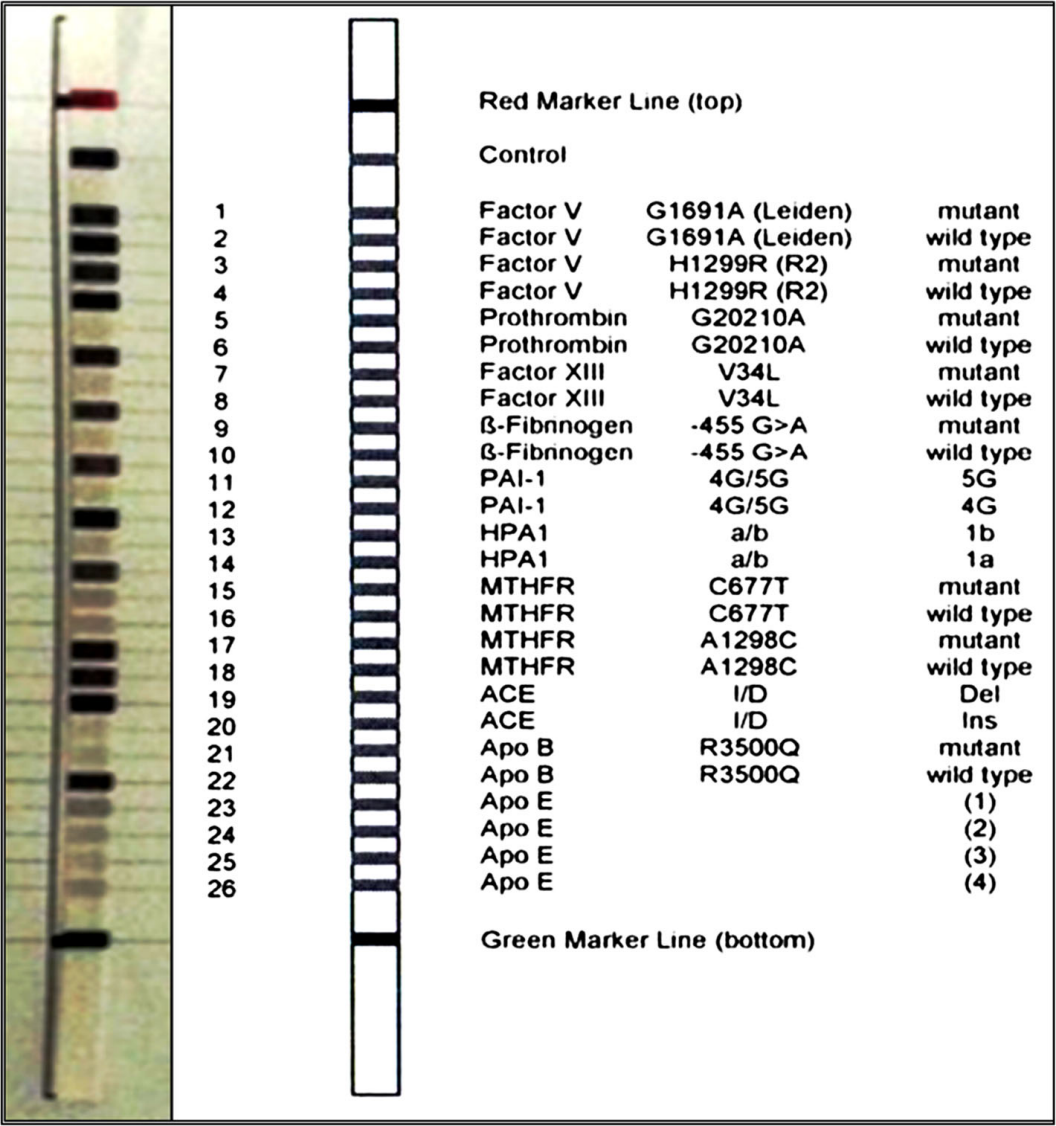
Design of CVD strip assay test used in the current study (Ref 4-240). The left strip represents an example of the genotype results of a case in the current study

DNA was isolated from whole blood within 2 h from collection using ABIOpureTM Total DNA (version 2.0) (AllianceBio, Catalog no. M501DP100) following the protocol supplied by the manufacturer. DNA concentration and purity at the absorbance ratio 260/280 nm were determined by NanoDrop ND-1000 spectrophotometer (NanoDrop Tech., Inc. Wilmington, DE, USA). Amplification was performed using biotinylated ready-made primers enclosed in CVD StripAssay kit (ViennaLab Labordiagnostika GmbH, Vienna, Austria) [40]; Fig. 1. Polymerase chain reaction (PCR) was performed in a 25-μl reaction volume containing 15 μl amplification mix, 5 μl of Taq DNA polymerase (0.2 U/μl) in the Taq dilution buffer, and 200 ng DNA template. It was carried out in a T-Professional Basic, Biometra PCR System (Biometra, Goettingen, Germeny) at 94 °C for 2 min as pre-PCR, then thermocycling at 94 °C for 15 s, 58 °C for 30 s, and 72 °C for 30 s (35 cycles), with subsequent final extension at 72 °C for 3 min and hold at 4 °C. Amplification products were analyzed by gel electrophoresis (2.5% agarose gel). The following fragment lengths were detected: 134, 165, 173, 202, 223, 254, 283, 324 bp.

### Reverse multiplex hybridization

Hybridization of amplification products with saline sodium phosphate EDTA (SSPE) buffer containing 0.5% sodium dodecyl sulfate (SDS) for 30 min was performed to the test strips containing allele-specific oligonucleotide probes immobilized as an array of parallel lines. The strip assays cover 12 variants in the following order: *FV* 1691G > A (Leiden), *FV* H1299R (R2), *Prothrombin* 20210G > A, *Factor XIII* V34L, *β-fibrinogeen* -455G > A, *PAI-1* 4G/5G, *HPA1* a/b, *MTHFR* 677C > T, *MTHFR* 1298A > C, *ACE* I/D, *Apo B* R3500Q, *Apo E* ε1/ε2/ε3/ε4, Fig. 1. Following hybridization steps, the bounded biotinylated sequences on the strips were washed with prewarmed Stringent Wash Solution A (SSPE buffer containing 0.1% SDS) in a microplate thermo-shaker (PST-60HL, Biosan, Riga, Latvia). Then incubated with a conjugate solution containing streptavidin-alkaline phosphatase at room temperature for 15 min. Repeated washing steps with Wash Solution B containing 0.05% sodium azide (NaN_3_) was done. Finally color development was performed using color substrates containing nitro blue tetrazolium (NBT) and 5-bromo-4-chloro-3-indolyl phosphate (BCIP) in the dark. Genotype interpretation was determined using the enclosed Collector^TM^ sheet with certain scale. Positive and negative control lines were checked for strip validation; Fig. 1. Positively stained lines were visually noted for each polymorphic position by two independent coauthors and signal pattern of bands were translated into schematic results using stripassay® online calculator [41]; (Additional file 2: Figure S1).

### Statistical analysis

Data were managed using the “Statistical Package for the Social Sciences (SPSS) for windows” software (version 20.0) with the aid of Deducer for R-2.15.0 and Microsoft excel 2016. Hardy-Weinberg equilibrium in patients and controls was estimated using the Online Encyclopedia for Genetic Epidemiology (OEGE) software [42]. Pairwise linkage disequilibrium (LD) based on the genotyping data was computed using Haploview software version 4.1. Five genetic association models were used; allelic model, homozygote and heterozygote comparison, dominant and recessive models [43]. Categorical variables were compared using the chi-square (*χ*2) or Fisher’s exact tests where appropriate, while the Student̕ s *t* test was used to compare continuous variables between two groups in case the data distribution was concordant with normal distribution (Shapiro-Wilk test) and after checking variance homogeneity (Levene test). If the data did not meet the criteria mentioned above, the non-parametric Mann Whitney-U (MW) and Kruskal-Wallis tests were applied. Spearman’s rank correlation coefficient was used for correlation analysis. A two-tailed *p*-value of < 0.05 was considered statistically significant. Logistic regression has been performed to adjust for significant risk factors using the backward method. For clustering the study participants according to gene-gene and gene-environment interaction, Detrended Correspondence Analysis (DCA) was implemented in PC-ORD version 5, using the default number of segments 26 and rescaling axes [44].

## Results

### Baseline characteristics of the study population

The general clinical and biochemical characteristics of the study groups are shown in Table 1. A total of 23 patients (17 men and 6 women) had undergone coronary angiography, while 34 apparently healthy subjects were assigned to the control group. There was no significant difference in the frequency of traditional risk factors of cardiovascular disease between patients and controls (as gender, obesity, family history of CAD, and diabetes mellitus). None of the controls were smokers or hypertensive. However, CAD patients had significantly higher glucose levels and worse lipid profile than controls (*p* ≤ 0.001). A higher proportion of patients had significantly over three traditional risk factors compared to controls (60.9 and 5.9%, respectively). Coronary angiography revealed that fifth of patients (21.7%) had no occlusion and only 15 out of 23 patients had significant stenosis (≥50% occlusion); 40% (6/15) of whom had complete luminal occlusion. In addition, nearly two-thirds (10/18) of patients with luminal occlusion, had multivessel disease.

**Table 1:** Clinical and biochemical characteristics of CAD patients and controls

Characteristics	Controls (*n* = 34)	Patients (*n* = 23)	*p* value	OR (95% CI)
Age, mean (y)	54.7 ± 10.1	54.0 ± 10.1	0.810	
Gender				
Females	16 (47.1)	6 (26.1)	0.166	*Reference*
Males	18 (52.9)	17 (73.9)		2.51 (0.79–7.94)
BMI, kg/m^2^	27.7 ± 1.86	28.1 ± 2.07	0.435	
Obesity	6 (17.6)	5 (21.7)	0.742	1.29 (0.34–4.88)
Smoking	--	13 (56.5)		
FH CAD	12 (35.3)	6 (26.1)	0.567	0.64 (0.20–2.07)
Hypertension	--	10 (43.5)		
Diabetes	12 (35.3)	7 (30.4)	0.780	0.82 (0.25–2.49)
Dyslipidemia	--	19 (82.6)		
Biochemical data				
FBS (mg/dl)	99.4 ± 25.7	145.6 ± 62.2	**<0.001**	
TC (mg/dl)	170 ± 18.4	207 ± 61.5	**0.001**	
TG (mg/dl)	103 ± 33.1	155 ± 54.4	**<0.001**	
LDL-c (mg/dl)	74.9 ± 12.6	147 ± 66.3	**<0.001**	
HDL-c (mg/dl)	48.6 ± 8.3	37.8 ± 11.4	**<0.001**	
No of risk factors				
≤ 3	32 (94.1)	9 (39.1)	**<0.001**	*Reference*
> 3	2 (5.9)	14 (60.9)		**24.8 (4.75–130)**
Premature CAD	--	9 (39.1)		
Previous events	--	18 (78.3)		
Stroke	--	1 (4.3)		
Lesion type				
100% Normal	--	5 (21.7)		
< 50% Occlusion	--	3 (13.0)		
50- 99% Occlusion	--	9 (39.2)		
100% Occlusion	--	6 (26.1)		
Lesion site				
Single VD	--	8 (34.8)		
Two VD	--	4 (17.4)		
Three VD	--	6 (26.1)		
CAD severity				
Gensini score	--	14 (1.5–62)		
Vessel score	--	1.0 (0.0–3.0)		
Echo findings				
Ejection fraction	–	57.4 ± 7.98		
Mild DD	--	20 (87.0)		
SWMA	--	9 (39.1)		

Values are shown as a number (percentage), mean ± standard deviation, or median (quartiles). *CAD* coronary artery disease; *OR* (*95% CI*), Odds ratio (95% confidence interval); *FH* family history, *FBS* fasting blood sugar, *TC* total cholesterol, *TG* triglycerides, *LDL-c* low density lipoprotein-cholesterol, *HDL-c* high density lipoprotein-cholesterol; Risk factors, Age (men ≥ 45 y and women ≥ 55 y), family history of premature coronary artery disease, hypertension, cigarette smoking, diabetes, hypercholesterolemia, low HDL cholesterol <40 mg/dl, hypertriglyceridemia > 200 mg/dl, and obesity; Premature coronary artery disease, <55 years in males and <65 years in females; previous events, previous acute coronary ischemic events; *VD* vessel disease (defined as luminal narrowing of > 70%); Gensini score to asses disease severity; *DD* diastolic dysfunction; *SWMA*, systolic wall motion abnormalities. *P* value was generated by using Fisher’s exact test, and Student’s t tests. The bold data are statistically significant at *p* < 0.05

### Genotype analysis of thrombotic and atherosclerotic variants

The observed genotype distribution of thrombotic and atherosclerotic genes were in agreement with Hardy-Weinberg equilibrium in both patients and controls (*p* > 0.05). However, genotype frequencies of *MTHFR* 667C > T in the CAD group and *ACE* I/D in the control group deviated significantly from the HWE (*p* = 0.041 and 0.001, respectively), possibly due to low sample size.

Among the studied genes, both coagulation factor II (rs1799963) and ApoB100 (rs5742904) variants were normal in all patients and controls, thus they were excluded from further analyses. As shown in Tables 2 and 3, the univariate model indicated that the 5 SNPs (1) rs1800595 (*FVR*), (2) rs1801133 (*MTHFR*), (3) rs5918 (*HPA-1*), (4) rs1799752 (*ACE*), and (5) rs7412 and rs429358 (*ApoE*) were significantly associated with CAD disease under different genetic association models. The heterozygote genotype of *FVR* 4070A > G was significantly higher in patients than controls (*p* = 0.024) with OR (95% CI) of 4.8 (1.2 to 18.3) and 3.8 (1.12–13.5) under heterozygote comparison and allelic models, respectively. The mutant homozygote TT genotype of *MTHFR* 667C > T also showed higher liability of CAD under homozygote comparison and allelic models. Carriers of the *HPA-1* b allele were more frequent among patients (0.26) compared to control group (0.12), (*p* = 0.048). *ACE* D/D genotype and D allele were more prevalent in CAD patients with OR showing higher susceptibility to develop CAD. Whereas, *ApoE* E2 variant showed protection against developing CAD (0.32 in controls compared to 0.13 in patients group, *p* = 0.004), while E3 and E4 individuals were >3 times more liable to have the disease compared to E2 carriers. The adjusted odds ratio for conventional risk factors (gender, age, BMI, family history of heart disease, diabetes, and obesity) further demonstrated that FVR*AG, *HPA-1* b/b, and ACE* DD genotypes are independent risk factors for occurrence of CAD.

**Table 2:** Genotype and allele frequencies of thrombotic gene variants in the study population

SNP	Genotypes	Controls (*n* = 34)	Patients (*n* = 23)	*P* values	Crude OR (95% CI)	Adjusted OR (95% CI)
*FV: Leiden*	Normal	32 (94.1)	20 (87.0)	0.384	1.0	1.0
1691G > A	Hetero	2 (5.9)	3 (13.0)		2.4 (0.3–15.6)	3.08 (0.3–26.3)
	*p* HWE	0.859	0.737			
	MAF (A)	0.03	0.07	0.359	0.4 (0.05–3.1)	
*FV: R2*	Normal	30 (8 8.2)	14 (60.9)	**0.024**	1.0	1.0
H1299R	Hetero	4 (11.8)	9 (39.1)		**4.8 (1.2–18.3)**	**7.1 (1.49–33.6)**
4070A > G	*p* HWE	0.715	0.243			
	MAF (G)	0.06	0.20	**0.024**	**3.8 (1.12–13.5)**	
*FII: PTH*	Normal	34 (100)	23 (100)	NA	NA	
20210G > A	*p* HWE	NA	NA			
*FXIII*	Normal	30 (88.2)	17 (73.9)	0.287	1.0	1.0
V34L	Hetero	4 (11.8)	6 (26.1)		2.6 (0.6–10.7)	2.29 (0.48–1.5)
	*p* HWE	0.715	0.471			
	MAF (T)	0.06	0.13	0.185	0.4 (0.09–1.6)	
*MTHFR*	Normal	22 (64.7)	12 (52.2)	**0.017**	1.0	1.0
677C > T	Hetero	12 (35.3)	6 (26.1)		0.9 (0.2–3.1)	0.82 (0.21–3.3)
	Homo	0 (0.0)	5 (21.7)		**19 (1.0–388)**	
	Dominant model	12 (35.3)	11 (47.8)		1.6 (0.5–4.9)	
	*p* HWE	0.211	**0.041**			
	MAF (T)	0.18	0.35	**0.037**	**2.4 (1.04–5.9)**	
*MTHFR*	Normal	10 (29.4)	8 (34.8)	0.648	1.0	1.0
1298A > C	Hetero	16 (47.1)	8 (34.8)		0.6 (0.1–2.2)	0.87 (0.21–3.6)
	Homo	8 (23.5)	7 (30.4)		1.1 (0.2–4.3)	1.46 (0.33–6.4)
	Dominant model	24 (70.6)	15 (65.2)		0.7 (0.2–2.4)	
	*p* HWE	0.745	0.146			
	MAF (C)	0.47	0.48	0.935	0.9 (0.4–2.1)	
*PAI-1*	4G/4G	10 (29.4)	7 (30.4)	0.698	1.0	1.0
4G/5G	4G/5G	18 (52.9)	10 (43.5)		0.7 (0.2–2.7)	1.15 (0.24–5.3)
	5G/5G	6 (17.6)	6 (26.1)		1.4 (0.3–6.3)	1.36 (0.27–6.8)
	Dominant model	24 (70.5)	16 (69.6)		0.9 (0.3–3.0)	
	*p* HWE	0.667	0.536			
	MAF (5G)	0.44	0.48	0.696	0.8 (0.4–1.8)	

*FV* coagulation factor 5, *PTH* Prothrombin, *FXIII* coagulation factor 13, *MTHFR* Methylenetetrahydrofolate reductase, *PAI-1* plasminogen activator inhibitor-1

Fisher’s Exact and Chi-square tests were used; adjusted by potential confounders (gender, age, BMI, FH, DM, obesity). Bold values indicate statistically significant at *p* < 0.05

**Table 3:** Genotype and allele frequencies of atherosclerotic gene variants in the study population

SNP	Genotypes	Controls (*n* = 34)	Patients (*n* = 23)	*P* values	Crude OR (95% CI)	Adjusted OR (95% CI)
*FGB*	Normal	22 (64.7)	12 (52.2)	0.636	1.0	1.0
-455G > A	Hetero	10 (29.4)	9 (39.1)		1.6 (0.5–5.2)	0.28 (0.02–3.1)
	Homo	2 (5.9)	2 (8.7)		1.8 (0.2–14.7)	1.65 (0.1–20.2)
	Dominant model	12 (35.3)	11 (47.8)		1.6 (0.5–4.9)	
	*p* HWE	0.557	0.866			
	MAF (A)	0.21	0.28	0.344	1.5 (0.6–3.6)	
*HPA-1*	a/a	26 (76.5)	13 (56.5)	0.112	1.0	1.0
a/b	a/b	8 (23.5)	8 (34.8)		2.0 (0.6–6.5)	
	b/b	0 (0.0)	2 (8.7)		9.8 (0.4–21.9)	**4.0 (1.4–6.4)**
	Dominant model	8 (23.5)	10 (43.5)		2.5 (0.7–7.8)	
	*p* HWE	0.436	0.638			
	MAF (b)	0.12	0.26	**0.048**	2.6 (0.9–7.1)	
*ACE*	I/I	6 (17.6)	2 (8.7)	**<0.001**	1.0	1.0
I/D	I/D	26 (76.5)	9 (39.1)		1.0 (0.1–6.1)	2.79 (0.3–29.7)
	D/D	2 (5.9)	12 (52.2)		**18 (2.0–161)**	**88.3 (4.3–179)**
	Dominant model	28 (82.4)	21 (91.3)		2.3 (0.4–12.2)	
	*p* HWE	0.001	0.866			
	MAF (D)	0.44	0.72	**0.003**	**3.2 (1.4–7.1)**	
*ApoB*	Normal	34 (100)	23 (100)	NA	NA	
R3500Q	*p* HWE	NA	NA			
*ApoE*	E2/E2	0 (0.0)	1 (4.3)	**0.009**	1.0	
E2/E3/E4	E2/E4	2 (5.9)	1 (4.3)		0.2 (0.0–8.8)	
	E2/E3	20 (58.8)	3 (13.0)		0.05 (0.0–1.6)	
	E3/E3	10 (29.4)	15 (65.2)		0.4 (0.01–13.2)	
	E3/E4	2 (5.9)	3 (13.0)		0.4 (0.01–16.8)	
	*p* HWE^a^	0.060	0.550			
	E3	0.61	0.78	**0.004**	1.0	
	E2	0.32	0.13		**0.08 (0.04–0.18)**	
	E4 (MAF)	0.06	0.09		0.61 (0.2–1.87)	

*FGB* fibrinogen beta polypeptide chain, *HPA-1* human platelet antigen 1, *ACE* angiotensin converting enzyme, *ApoB* Apolipoprotein B-100, *ApoE* apolipotprotein E, ^a^The number of degrees of freedom for the chi-square is equal to 3 for three allele systems and 1 for two allele systems. *MAF* minor allele frequency. Fisher’s Exact and Chi-square tests were used. Adjusted OR by potential confounders (gender, age, BMI, FH, DM, obesity). Bold values indicate statistically significant at *p* < 0.05

**Fig. 2:**
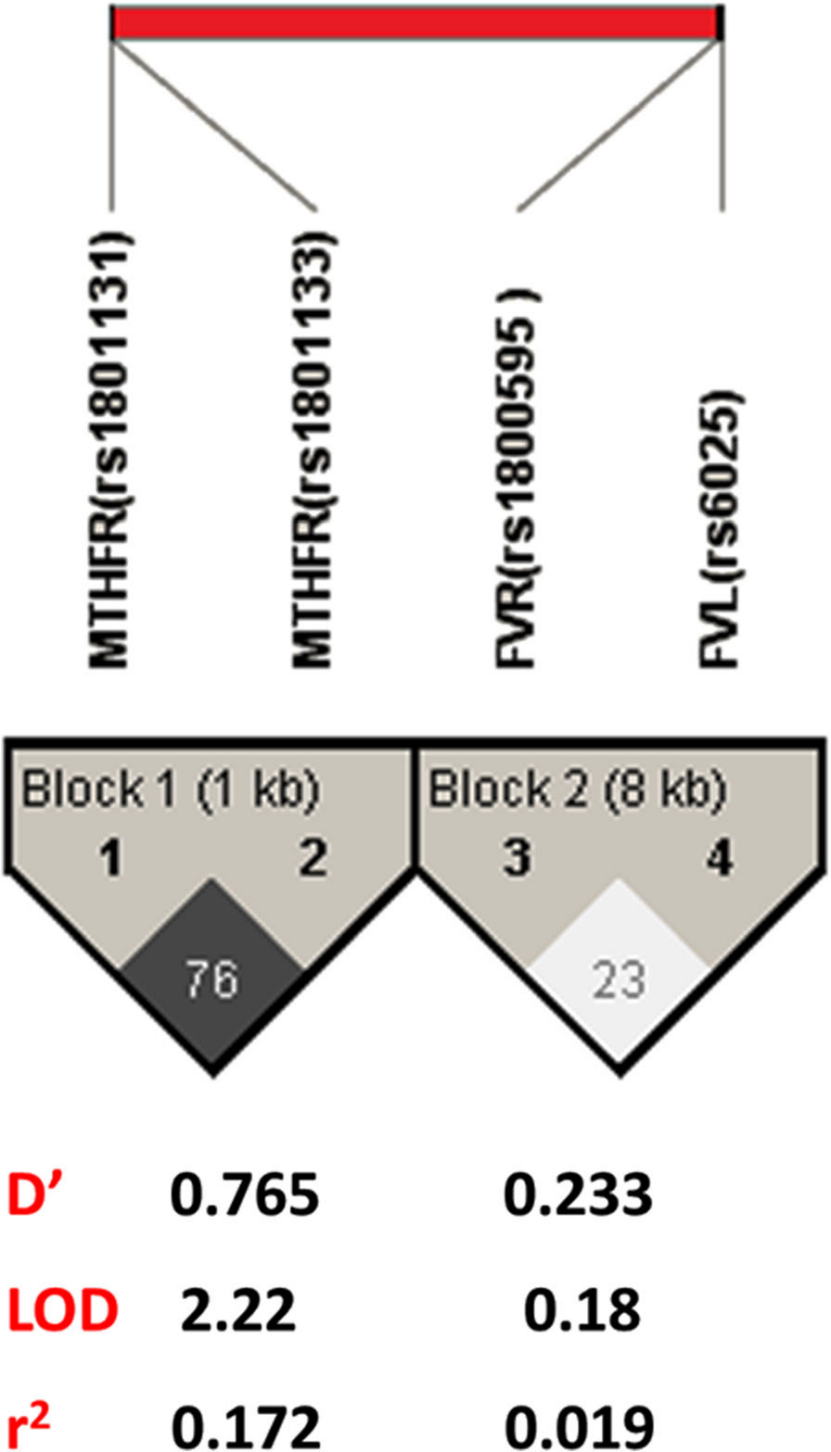
Estimated linkage disequilibrium and location of haplotype blocks. LD plots with 1 Kb and 8 Kb between marks are contained within black triangles in the figure, and markers order are outside these triangles. Evidence for LD is shown as different shades (white for low D’ and dark gray for high D’). The haploblocks were identified using a confidence interval algorithm in haploview program version 4.1

Among the studied genes, four polymorphisms are located on chromosome 1: *FVL* (rs6025), *FVR* (rs1800595), *MTHFR* (rs1801133), and *MTHFR* (rs1801131). Haplotype analysis showed the presence of two blocks; block 1 included 667C > T and 1298A > C SNPs of *MTHFR* gene and block 2 involving *F5: Leiden* 1691G > A and *F5: R2* 4070A > G. The marker locations and LD block structure of the haplotype blocks are shown in Fig. 2. Our analysis revealed a significant difference in the distribution of GA haplotype in block 2 between cases and controls (*p* = 0.039), Table 4.

**Table 4:** Haplotype frequencies and genotype combinations of both *F5: Leiden* 1691G > A with *F5: R2* 4070A > G, and *MTHFR* 667C > T with 1298A > C polymorphisms in CAD patients and controls

Haplotypes^a^	Overall frequency	Controls (*n* = 34)	Patients (*n* = 23)	*P* values	OR (95% CI)
Genotype combination: *MTHFR* 667C > T/1298A > C					
CC-AA	11 (19.3)	8 (23.5)	3 (13.0)	0.150	*Reference*
CT-AA	3 (5.3)	2 (5.9)	1 (4.3)		1.3 (0.08–20.7)
TT-AA	4 (7.0)	0 (0.0)	4 (17.4)		21.8 (0.9–523)
CC-AC	9 (15.8)	6 (17.6)	3 (13)		1.3 (0.19–9.08)
CT-AC	14 (24.6)	10 (29.4)	4 (17.4)		1.06 (0.18–6.2)
TT–AC	1 (1.8)	0 (0.0)	1 (4.3)		7.2 (0.23–225)
CC-CC	14 (24.6)	8 (23.5)	6 (26.1)		2.0 (0.3–10.9)
CT-CC	1 (1.8)	0 (0.0)	1 (4.3)		7.2 (0.23–225)
Genotype combination: *FVL* 1691G > A/*FVR* 4070A > G					
GG-AA	41 (71.9)	28 (82.4)	13 (56.5)	0.080	*Reference*
GG-AG	11 (19.3)	4 (11.8)	7 (30.4)		3.7 (0.9–15.18)
GA–AA	3 (5.3)	2 (5.9)	1 (4.3)		1.07 (0.08–12.9)
GA-AG	2 (3.5)	0 (0.0)	2 (8.7)		10.5 (0.47–235)
Block 1: *MTHFR* 667C > T/1298A > C					
CC	0.446	45.9	42.8	0.7437	
CA	0.308	36.5	22.4	0.1114	
TA	0.218	16.5	29.8	0.0926	
TC	0.027	1.1	5	0.2134	
Block 2: *FVL* 1691G > A/*FVR* 4070A > G					
GA	0.856	91.2	77.4	**0.0397**	
GG	0.1	5.9	16.1	0.075	
AA	0.03	2.9	3	0.9767	
AG	0.014	0	3.5	0.121	

*MTHFR*, Methylenetetrahydrofolate reductase; *FV*, coagulation factor 5

^a^Haploview version 4.1 was used for data analysis. Bold values indicate statistically significant at *p* < 0.05

### Interaction of both gene variants and traditional risk factors with disease risk

**Fig. 3:**
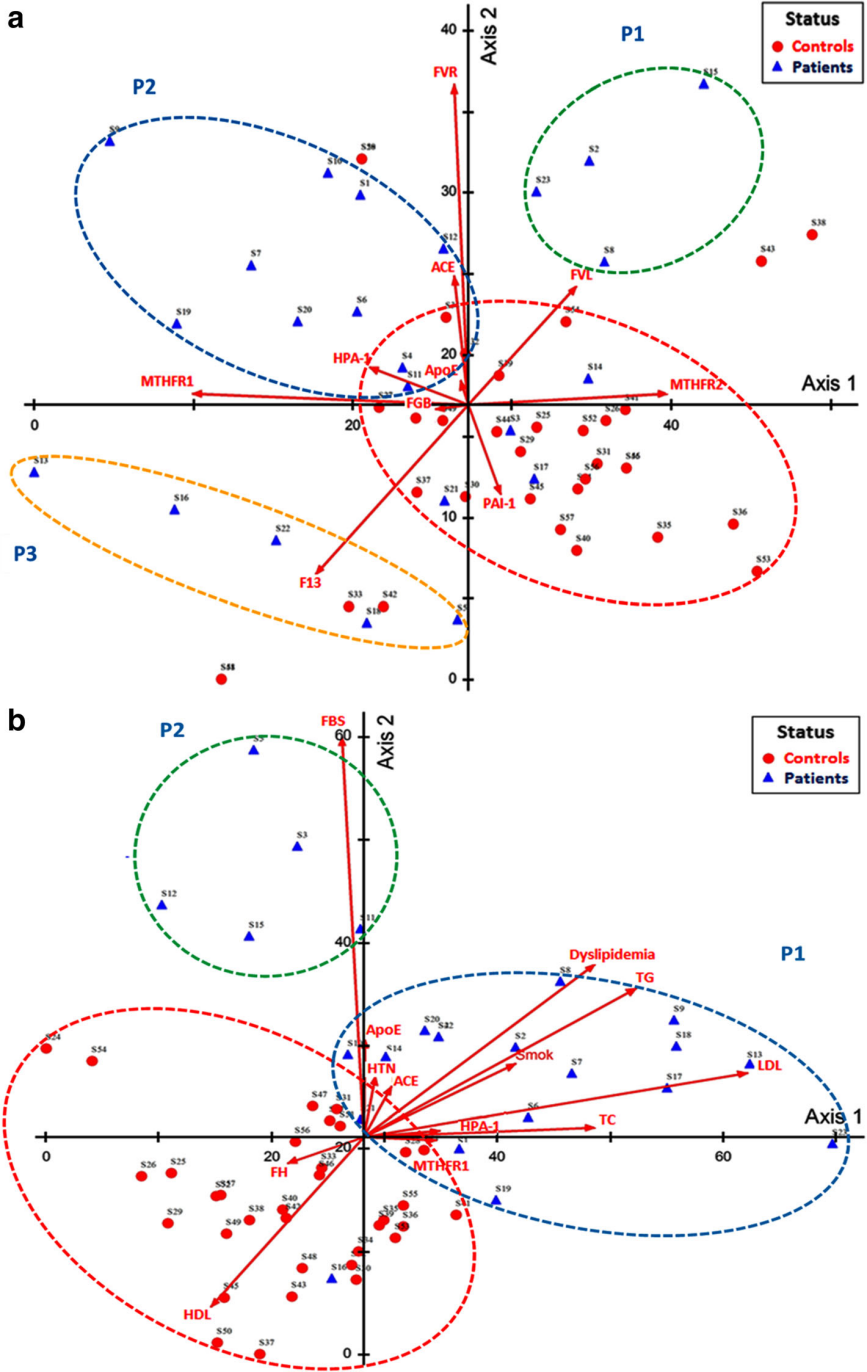
Detrended correspondence analysis ordination plots showing the distribution of patient and control samples. DCA was done to show the relations among subject groups and genes (**a**) along with other environmental risk factors (**b**). DCA was done after rescaling axes. Red circle surrounds a control group while other colored circles for patient groups. Results were plotted on Axes 1 and 2

Genotype frequencies of variants in patients and controls stratified by cardiovascular risk factors are illustrated in Additional file 1: Tables S2 and S3. Significant difference of frequencies of SNPs was observed with gender, obesity and diabetes. Multivariate analysis using an ordination method was used for data clustering, Fig. 3. Analysis of the whole gene variants showed a tendency of the studied population to cluster into 4 groups; one control and three patient groups. Among patients’ clusters, *FVR* was the most effective associated gene variant in most of our CAD patients, combined with the *FVL* variant in P1 cluster and with both *ACE* and *MTHFR* 667C > T in P2 cluster. Whereas, the third patient group (P3) was mostly affected by both *MTHFR* 667C > T and *F13* V89L mutations, Fig. 3a. With the intersection of genes with confounders and traditional risk factors, more discrimination between patients and controls was revealed in Fig. 3b. HDL and family history were the most prominent factors in the control group. Patients were clustered into two groups showing more effect of traditional risk factors than genes; P1 group was mostly affected by dyslipidemia, LDL, total triglyceride, total cholesterol, smoking, and hypertension, with little contributing effect of gene variants (*HPA-1*, *ACE*, *ApoE*, and *MTHFR*1), while P2 cluster was mostly affected by their fasting blood sugar levels and *ApoE* variant.

Correlation analysis between gene variants and risk factors supported the association between these genes and lipid profile, Additional file 1: Table S4. Four thrombotic and four atherosclerotic gene variants were significantly associated with dyslipidemia and lipid profile; *FVR* (4070A > G) with higher total triglyceride (*p* = 0.004) and lower HDL (*p* = 0.030), *F13A1* (V35L) and *HPA-1* (a/b) with higher LDL (*p* = 0.047 and 0.024, respectively), *MTHFR* (667C > T) with total cholesterol (*p* = 0.004), and *ApoE* with triglyceride (*p* = 0.002) and LDL (*p* = 0.017). In addition, similar to DCA plot results, *ApoE* was also positively correlated with fasting blood sugar levels (r = 0.267, *p* = 0.044). However, *HPA-1* (a/b) variant showed significant association with patients’ age (r = -0.352, *p* = 0.007); youngest age (35.5 ± 10.6) in mutant homozygote individuals, intermediate age (51.3 ± 9.5) in heterozygotes, and later age at onset (56.7 ± 9.0) in normal homozygote carriers (*p* = 0.016).

### Association of traditional risk factors with disease phenotype

Multivessel disease was more frequent among males (*p* = 0.043) and smokers (*p* = 0.020). Males also had a higher gensini score (*p* = 0.048) with OR (95% CI) of 15 (1.2–18.5). CAD patients who had experienced previous acute coronary ischemic events were two times more likely to have significant occlusion >50% (*p* = 0.033). No association was observed between other traditional risk factors and the degree of CAD at coronary angiography, Table 5. In addition, none of the risk factors showed association with any other clinical characteristics as premature CAD, previous acute coronary ischemic events, echocardiography findings (ejection fraction, diastolic dysfunction, and systolic wall motion abnormalities) in CAD patients (data not shown).

**Table 5:** Association between traditional risk factors and clinical characteristics of CAD patients

Risk factor	Gensini score		*P* value	Occlusion		*P* value	No of BVs		*P* value
	Low	High		<50%	≥50%		SVD	MVD	
Number	14	9		8	15		8	10	
Gender									
Females	6 (42.9)	0 (0.0)	**0.048**	2 (25.0)	4 (26.7)	1.00	5 (62.5)	1 (10)	**0.043**
Males	8 (57.1)	9 (100)		6 (75.0)	11 (73.3)		3 (37.5)	9 (90)	
Obesity	2 (14.3)	3 (33.3)	0.343	0 (0.0)	5 (33.3)	0.122	2 (25)	3 (30)	1.00
Smoking	6 (42.9)	7 (77.8)	0.197	4 (509	9 (60.0)	0.685	2 (25)	8 (80)	**0.020**
FH CAD	2 (14.3)	4 (44.4)	0.162	2 (25.0)	4 (26.7)	1.00	1 (12.5)	4 (40)	0.314
Hypertension	7 (50.0)	3 (33.3)	0.669	3 (37.5)	7 (46.7)	1.00	5 (62.5)	4 (40)	0.637
Diabetes	4 (28.6)	3 (33.3)	1.00	2 (25.0)	5 (33.3)	1.00	3 (37.5)	3 (30)	1.00
Dyslipidemia	11 (79)	8 (88.9)	1.00	6 (75.0)	13 (86.7)	0.589	5 (62.5)	9 (90)	0.275
Premature CAD	5 (35.7)	4 (44.4)	1.00	3 (37.5)	6 (40)	1.00	3 (37.5)	4 (40)	1.00
Previous events	9 (64.3)	9 (100)	0.116	4 (50.0)	14 (93.3)	**0.033**	7 (87.5)	9 (90)	1.00

Data are presented as number (percentage). *CAD* coronary artery disease, *FH* family history; premature coronary artery disease, <55 years in males and <65 years in females; previous events, previous acute coronary ischemic events, *No of BVs* number of blood vessels, *SVD* single vessel disease, *MVD* multivessel disease. Fisher’s exact test was used. Bold values indicate statistically significant at *p* < 0.05

### Association of gene variants with disease phenotype

As shown in Table 6, CAD patients carrying mutant allele of *FVR* polymorphism had a lower genseni score (*p* = 0.04) with OR (95% CI) of 0.09 (0.009–0.96) (r = -0.460, *p* = 0.027), and lower risk of significant occlusion ≥ 50% (*p* = 0.023) with OR (95% CI) of 0.08 (0.01–0.64) (r = -0.536, *p* = 0008). In addition, *MTHFR* 1298A > C mutant homozygotes had a better genseni score (*p* = 0.035).

**Table 6:** Association between SNP genotypes and clinical characteristics of CAD patients

Gene SNP	G	Gensini score		*P* value	Occlusion		*P* value	No of BVs		*P* value
		Low	High		<50%	≥50%		SVD	MVD	
Number		14	9		8	15		8	10	
*FVL*	N	11 (78.6)	9 (100)	0.253	7 (87.5)	13 (86.7)	1.00	7 (87.5)	9 (90)	1.00
	H	3 (21.4)	0 (0.0)		1 (12.5)	2 (13.3)		1 (12.5)	1 (10)	
*FVR*	N	6 (42.9)	8 (88.9)	**0.040**	2 (25)	12 (80)	**0.023**	4 (50)	9 (90)	0.118
	H	8 (57.1)	1 (11.1)		6 (75)	3 (20)		4 (50)	1 (10)	
*F13A1*	N	12 (85.7)	5 (55.6)	0.162	6 (75)	11 (73.3)	1.00	7 (87.5)	6 (60)	0.314
	H	2 (14.3)	4 (44.4)		2 (25)	4 (26.7)		1 (12.5)	4 (40)	
*MTHFR 1*	N	8 (57.1)	4 (44.4)	0.558	4 (50)	8 (53.3)	0.962	6 (75)	5 (50)	0.234
	H	4 (28.6)	2 (22.2)		2 (25)	4 (26.7)		2 (25)	2 (20)	
	M	2 (14.3)	3 (33.3)		2 (25)	3 (20)		0 (0.0)	3 (30)	
*MTHFR 2*	N	4 (28.6)	4 (44.4)	**0.035**	4 (50)	4 (26.7)	0.250	2 (25)	4 (40)	0.167
	H	3 (21.5)	5 (55.6)		1 (12.5)	7 (46.7)		2 (25)	5 (50)	
	M	7 (50)	0 (0.0)		3 (37.5)	4 (26.7)		4 (50)	1 (10)	
*PAI-1*	N	5 (35.7)	2 (22.2)	0.730	2 (25)	5 (33.3)	0.660	3 (37.5)	3 (30)	0.869
	H	6 (42.9)	4 (44.4)		3 (37.5)	7 (46.7)		3 (37.5)	5 (50)	
	M	3 (21.4)	3 (33.3)		3 (37.5)	3 (20)		2 (25)	2 (20)	
*FGB*	N	7 (50)	5 (55.6)	0.490	3 (37.5)	9 (60)	0.583	5 (62.5)	5 (50)	0.799
	H	5 (35.7)	4 (44.4)		4 (50)	5 (33.3)		2 (25)	4 (40)	
	M	2 (14.3)	0 (0.0)		1 (12.5)	1 (6.7)		1 (12.5)	1 (10)	
*HPA-1*	N	7 (50)	6 (66.7)	0.595	4 (50)	9 (60)	0.851	6 (75)	6 (60)	0.610
	H	6 (42.9)	2 (66.7)		3 (37.5)	5 (33.3)		2 (25)	3 (30)	
	M	1 (7.1)	1 (22.2)		1 (12.5)	1 (6.7)		0 (0.0)	1 (10)	
*ACE*	N	1 (7.1)	1 (11.1)	0.829	1 (12.5)	1 (6.7)	0.894	0 (0.0)	1 (10)	0.287
	H	5 (35.7)	4 (44.4)		3 (37.5)	6 (40)		2 (25)	5 (50)	
	N	8	4 (44.4)		4 (50)	8 (53.3)		6 (75)	4 (40)	
*ApoE*	2/2	0 (0.0)	1 (11.1)	0.492	0 (0.0)	1 (6.7)	0.658	0 (0.0)	1 (10)	0.834
	2/3	2 (14.3)	1 (11.1)		1 (12.5)	2 (13.3)		1 (12.5)	1 (10)	
	2/4	1	0 (0.0)		1 (12.5)	0 (0.0)		0 (0.0)	0 (0.0)	
	3/3	10	5 (55.6)		5 (62.5)	10 (66.6)		6 (75)	7 (70)	
	3/4	1	2 (66.7)		1 (12.5)	2 (13.3)		1 (12.5)	1 (10)	

Data are presented as number (percentage). *CAD* coronary artery disease, *SNP* single nucleotide polymorphism, *G* genotype, *N* normal, *H* heterozygote, *M* mutant, *No of BVs* number of blood vessels, *SVD* single vessel disease, *MVD* multivessel disease, *FV* coagulation factor 5, *MTHFR* 1 and 2 Methylenetetrahydrofolate reductase (rs1801133) and (rs1801131), respectively, *PAI-1* plasminogen activator inhibitor-1, *FGB* fibrinogen beta polypeptide chain, *HPA-1* human platelet antigen 1, *ACE* angiotensin converting enzyme, *ApoE* apolipotprotein E. Fisher’s exact test was used. Bold values indicate statistically significant at *p* < 0.05. *PTH* (Prothrombin) and *ApoB* (Apolipoprotein B-100) gene variants were excluded from analysis as all patients were normal

Association analysis of gene variants with other clinical characteristics and echocardiography parameters showed *FVR* polymorphism to be inversely related to systolic wall motion abnormalities (r = -0.416, *p* = 0.048) and *F13A1* (V35L) SNP to be associated with lower ejection fraction in heterozygote compared to normal homozygote (*p* = 0.020) with moderate inverse correlation (r = -0.50, *p* = 0.015) (data not shown).

## Discussion

To our knowledge, this is the first study conducted to investigate the interaction between twelve atherosclerosis and thrombosis related gene variants and the environmental risk factors that could increase the susceptibility to coronary artery disease in Egyptian CAD patients. The previous studies have confirmed that understanding the gene–environment interactions is very important for the development of novel preventive and therapeutic approaches to reduce the risk of CAD [45–47].

In the current study *FVR* was the most variant associated with CAD patients, combined with the *FVL* variant to increase CAD risk in one of the patient clusters and with both *ACE* and *MTHFR* 667C > T in the second cluster. Because CAD is a multifactorial disorder, its genetic components may be a combined effect of a number of genes with each playing only a small role. Butt et al. [48] have reported that the predisposition imparted by individual genes may act independently or interact with other genes to result in an additive effect or a synergistic coeffect. The *FV:R2* A4070G variant causes amino acid replacement of histidine for arginine at the codon 1299 in the FV B domain. This polymorphism, which marked by the HR2 haplotype was reported to affect the plasma level of FV and contribute to the APC resistance [49]. Although the homo mutant genotype was absent in CAD patients and controls in the current work, this variant confers high susceptibility to CAD risk under the heterozygous and allelic models. Segers et al. [50] suggested that the reduced level of expression of *FV*: *R2* is the most likely explanation for the enhanced thrombophilic phenotype in *R2* carriers. Although there are no conclusive data available that associate the *R2* haplotype *per se* with a relevant increased risk of thrombosis (i.e. taken in consideration that *FV: Leiden* frequency was comparable between CAD patient and control groups in the current study), it was observed previously that male carriers of the *FV: R2* polymorphism, have increased circulating levels of FVIII [51]. Although there was no biochemical explanation has been given for this observation, it has been speculated that altered FV anticoagulant activity of *FV: R2* results in diminished the proteolytic control of FVIII levels by the protein C pathway [50]. Since increased levels of FVIII are an independent risk factor for the occurrence of venous thrombosis, this may explain the potential association between *FV: R2* and thrombosis. Nevertheless, we found that patients carrying the G allele unexpectedly displayed a mild phenotype (i.e. lower Genseni score and lower number of MVD) than wild-type genotype carriers. It seems therefore that the HR2 haplotype in our population preferentially increases the CAD susceptibility but correlates with mild disease severity. It should be kept in mind, however, that thrombosis is a multifactorial disease in which genetic and acquired risk factors interact dynamically. Therefore, the role that FV plays in the etiology of thrombosis is largely dependent on other factors that influence the impact of any altered FV phenotype [50] and the disease outcome depends on the sum of other multiple interacting factors determine the severity of the disease. This finding should be validated in future larger scale studies to be conclusive.

The mutant homozygote TT genotype of *MTHFR* 667C > T (rs1801133) was found to be more frequent among the studied CAD Egyptian patients (21.7%) and was not found in the controls. This variant showed significantly higher susceptibility for CAD under homozygote and allelic models and it was one of the most effective associated gene variants in combination with other variants for clustering our CAD patients by multivariate analysis (i.e. the combined occurrence of the *MTHFR* 677TT, *ACE* DD and *FVR* 4070AG genotypes seems to magnify the CAD risk in our population). *MTHFR* gene is located on chromosome 1p36.3 and contains more than hundred SNPs, the most studied variants are rs1801133 and rs1801131 that included in the current study. The 667C > T transition in exon 5 of the first SNP is resulting in a change of alanine to valine (A222V) near the FAD (flavin-adenine-dinucleotide) cofactor binding region, and the other variant caused by the point mutation of A to C at nucleotide 1298 of coding sequence leads to substitution of glutamine by alanine at the amino acid number 470 in the protein structure [52]. In silico tools predicted the former variant (i.e. 667C > T) in exon 5 to be deleterious in contrast to the later neutral one. This could explain why *MTHFR 667C > T*; was associated with CAD risk in our population as the enzyme with valine was shown previously to be associated with increased thermoliability and reduced enzymatic activity to about 30% if mutant homozygous and 65% if heterozygous [53]. This reduced activity resulting in hyperhomocysteinemia when folate status is low that has been linked to thrombosis and considered as a major independent risk factor for cardiovascular diseases [54]. Similar to our results, when Almawi et al. [55] studied *FV* 1691 G > A, *PT* 20210 G > A, and *MTHFR* 677 C > T gene variants in angiographically documented CAD, they found a strong association of hyperhomocysteinemia and homozygosity of the *MTHFR* 677 C > T, but not *FV: Leiden* or *PT* 20210 G > A mutations with CAD. The results of the present study are also in agreement with Neto et al. [56] who found the *MTHFR* 677 T/T genotype to predispose to premature atherosclerotic coronary artery disease, and with Yamada et al. [57] and Schürks et al. [58] who reported increased risk for MI and ischemic stroke, respectively. Otherwise, our findings were in contrast to Hsu et al. [59], Yilmaz et al. [60] and Caner et al. [61] who reported that the *MTHFR* C677T mutation was not associated with the risk of CAD or venous thrombosis among Chinese in Taiwan nor among Turkish patients, respectively. These contradictory results between different studies could be attributed to relatively small sample size, low frequency of gene variants and ethnic heterogeneity [50].

The plasminogen activator inhibitor-1 (PAI-1) is a 52 kDa glycoprotein encoded by the serine protease inhibitor (*SERPINE1*) gene [62]. It is secreted by endothelial cells and is stored and released from the platelets during activation to downregulate the fibrinolysis process by inhibiting plasminogen to plasmin conversion induced by either tissue-plasminogen activator or urokinase [63]. The current study functional insertion/deletion promoter variant at -675 position (rs1799889), has been associated with increased risk of atherosclerosis and thrombosis previously with inconsistent results [20, 64–67]. As both the -675 4G and 5G alleles have a binding site for a common transcription activator and the 5G allele has an additional binding site for a repressor, this leads to 5G allele is slightly less transcriptionally active than the 4G and subsequently is associated with lower PAI-1 plasma levels and lower risk of thrombus stabilization [68]. Our results showed no association between *PA1* 4G/5G and the studied sample of CAD patients. This is in line with a previous study on Egyptian population [69] and others [20, 65, 70, 71] that also showed lack of association of this risk variant with MI or CAD. A number of evidences could elucidate our findings; first, the pathogenesis of CAD is complex and multifactorial with multiple interacting environmental and genetic determinants; hence PAI-1 is influenced not only by the gene SNP, but also by environmental factors, such as blood sugar, insulin and triglyceride concentrations among others [66]. Considering this fact, in subgroup stratified analysis, the 4G/4G genotype has been shown to be associated with obesity under the dominant genetic model to be in concordance with Mertens et al. [72], who found increased PAI-1 levels are associated with increased visceral obesity due to its production by ectopic fat depots [73]. In addition, Fernandes and Sandrim [74], found that obese women with 4G/4G genotypes were at increased risk of thrombotic diseases. The 5G/5G genotype in our cases has shown to be protective in patients with positive family history for CAD, to be in line with Margaglione et al. [75]. Second, variability in plasma concentrations of PAI-1 has been reported in different ethnic groups worldwide. The 4G/5G variant has been shown to be the predominant effector in some cases [76–78], while in others environmental factors such as smoking are involved [79].

Human platelet antigens (HPA), platelet surface receptors, play a key role in the adhesion, activation, and aggregation of platelets. They are formed of two glycoprotein (GP) subunits (GPIIb/IIIa) [80]. The GPIIIa subunit is polymorphic [81]. One of the most studied GPIIIa variant, *HPA-1* a/b (c.176 T > C; Lys59Pro), also known as PIA1/A2, has been suggested as an important genetic key player in the CAD pathogenesis [82]. In the current study, our data demonstrated an association between *HPA-1* a/b polymorphism and the occurrence of CAD. We found a significant higher prevalence of *HPA-1* a/b and HPA b/b genotypes among CAD patients compared to controls. This was consistent with the studies of Weiss et al. [83] and Abboud et al. [84] which reported a strong association between the *HPA-1* b allele and genotypes and the risk of CAD. The mutant allele encodes proline instead of lysine at an amino acid position located near the ligand binding site [81]. Bennet et al. [85] showed no impact on ligand binding using static model system. Similarly, our *in silico* analysis demonstrated L59P mutation to be benign and tolerated. However, *in vitro* cell culture showed enhanced binding of both fibrinogen and von Willebrand factor (vWF) to the receptor. In the normal population, the *HPA-1*b phenotype results in increased platelet aggregation and increased fibrinogen binding, thus conferred an increased risk of MI [23, 86], especially in early onset heart disease [83], ischemic stroke [87] and resistance to aspirin [88]. In contrast, other studies reported the absence of any association between the *HPA-1*a/b missense mutation and the occurrence of coronary diseases [89–92]. This discrepancy could be explained by the finding of Zotz et al. [93] who reported that *HPA-1* a/b is a risk determinant in patients with already existing atheromatous burden, but not for the development of atheromata.

One of the most popular genetic risk factors in CAD is the *ACE* insertion/deletion (rs1799752) polymorphism [94]. The *ACE* gene is located on chromosome 17q23.3 with 26 exons and 25 introns spreading over about 2 Kb [95]. The presence (insertion) or absence (deletion) of an Alu repetitive element (287-bp repeat sequence) in intron 16 results in I and D alleles, respectively [92]. This common polymorphism accounts for 47% of variations within *ACE* gene [94]. The results of the current study showed that DD genotype could be a considerable risk factor for CAD susceptibility, particularly in females. There was an increased frequency of D allele in CAD patients (0.72) compared to controls (0.44). No association of the gene was observed with other cardiovascular risk factors highlighting more strong effect of *ACE* with CAD. In 1992, Cambien and his colleagues reported the earliest significant relationship between the DD homozygosity and the risk of CAD [96], and that was followed by the study of Morris et al. [97] who suggested that the DD genotype represents a robust risk factor for MI and sudden death compared to II genotype. In addition, DD genotype was associated with coronary artery spasm that is considered one of the mechanisms of MI [98] and increased risk of restenosis with atherosclerotic lesions after percutaneous transluminal angioplasty [99]. Similar findings were further reported in two meta-analysis studies conducted by Samani et al. [100] and Zhou et al. [101]. Furthermore, numerous studies implicated the DD homozygosity as a key risk factor in other cardiovascular diseases, including hypertension [102], coronary and carotid atherosclerosis [103], cardiomyopathy [104], peripheral arterial disease [105], ischemic stroke [106], and recurrent venous thromboembolism [107]. In contrast, Lindpaintner et al. [108] did not confirm the association between D variant and increased risk of CAD in a large population of American males, and this finding was consistent with that of ours as stratification analysis by gender revealed significant association among females only. ACE, the key regulator in the renin angiotensin system (RAS), has two main physiological functions; converts angiotensin I into a physiologically active peptide angiotensin II (Ang II) and degrades bradykinin, a potent vasodilator [105]. Ang II is the atherogenic component of RAS that increases vascular permeability, stimulates proliferation and migration of vascular smooth muscle cells, induces the expression of inflammatory mediators, and enhances the deposition of extracellular matrix [109]. Reduced bradykinin by ACE decreased endothelium-dependent vasodilators nitric oxide and prostacyclin thus controls blood pressure [103]. The circulatory ACE level is genetically determined by the I/D variant [105]. Individuals with DD genotype have a twofold increase in the serum ACE concentration in the normal population, with ID subjects having intermediate levels [110], leading to higher levels of circulating Ang II and vasoconstriction which are common in MI and hypertensive patients [95]. The results of these studies correlate with those of Suehiro et al. [111] which showed an association between D allele and higher expression of the *ACE* mRNA that may affect the renin-angiotensin system in local regions. The mechanisms underlying this variation in levels of circulating enzymes by the absence of the Alu repeat sequence within the intron in DD genotype still need to be elucidated.

Two apolipoprotein variants were examined in our study; *ApoB-100* R3500Q mutation and *ApoE* (rs7412 and rs429358). ApoB is the primary apolipoprotein of chyomicrons, VLDL and LDL, which is responsible for carrying fat molecules throughout the body to all tissue cells. The ApoB on the LDL particle acts as a ligand for LDL receptors. High levels of ApoB are the primary drivers of atheroma formation [112]. It is well established that ApoB100 levels are associated with coronary heart disease [113, 114]. Mutations in ApoB gene can cause familial hypercholesterolemia [115]. The R3500Q (rs5742904) mutation in *ApoB* was found to be a major determinant of LDL levels and coronary artery calcification in the Amish population [112]. Substitution of Arginine with Glutamine is believed to prevent proper folding of ApoB and thereby reducing the ability of LDL particle to bind to the LDL receptor [116]. R3500Q mutation was absent in our study population, possible due to low sample size or ethnic variations. This was consistent with population-based surveys which reported low frequency of R3500Q mutation in non-Amish population [117]. The *ApoB* gene mutations were not detected in Lebanon [118], Russia [119], Iran [26], and Turkey [120].

The other studied apolipoprotein *ApoE* is found in chylomicrons and intermediate density lipoprotein (IDL) that is essential for the normal catabolism of triglyceride-rich lipoprotein constituents and cholesterol metabolism [121]. The *ApoE* gene, mapped on chromosome 19q13.32 in a cluster with *ApoC1* and *ApoC*2, is highly polymorphic [122], with 3 major alleles according to the amino acids at positions 130 (rs429358; c.388 T > C) and 176 (rs7412; c.526C > T) in exon 4: *ApoE*2 (cys130/cys176), *ApoE*3 (cys130/arg176), and *ApoE*4 (arg130/arg176) (ensemble.org). The *ApoE* polymorphisms were found to affect mRNA transcription and result in increased plasma cholesterol and triglyceride with impaired clearance of lipids from the bloodstream [121]. Moreover, knockout mice lacking *ApoE* developed extreme hypercholesterolemia after a high-fat diet [123]. In the present study, E3 (rs429358*T, rs7412*C) was the predominant form of gene in both patients and controls followed by E2 variant (rs429358*T, rs7412*T) among the study population, E2 carriers showed protection against developing CAD compared to E3 and E4. Both E3/E3 and E3/E4 genotypes had higher disease risk more than three times when compared to E2 genotypes. *ApoE* variants were also significantly associated with abnormal triglyceride and LDL-c among our CAD patients. Similar to our findings, E3 isoform was the most frequent *ApoE* variant in several populations, including Americans [124], Indians [125], Turkish [126], and Saudians [127], thus is considered the wild type *ApoE* genotype. The presence of E4 isoform (rs429358*C, rs7412*C) was previously associated with increased risk of CAD and mortality [128], atherosclerosis [129], and ischemic cerebrovascular disease [130]. Further meta-analyses studies showed *ApoE2* allele to be protective while E4 allele rendered higher susceptibility to heart diseases [119, 131–133]. Although these allelic forms differ from each other by only one or two amino acids at 2 sites, these differences alter the ApoE protein structure and function, and thereby have significant physiological consequences [134]. E2 variant with 2 cystienes binds poorly to LDL cell surface receptor compared to E3 and E4, thus can increase the LDLR number, and lowering cholesterol level [122]. On the other hand, E4 isoform with two arginine amino acids exhibits enhanced transfer from HDL to TG-rich lipoproteins, promoting hepatic remnant clearance by ApoE receptors and decreasing LDLR, thereby increasing circulating cholesterol levels [122]. The same variant has also been associated with increased calcium ion levels and apoptosis following mechanical injury [135].

## Conclusions

With the caveat that our sample size for this analysis was low, and the current analyses are likely to have been underpowered, our study confirms that CAD development requires more complex interactions among several risk factors [89], including genetic and environmental one. More specifically, it has been found that five variants; rs1800595 (*FVR*), rs1801133 (*MTHFR*), rs5918 (*HPA-1*), rs1799752 (*ACE*), and rs7412 and rs429358 (*ApoE*) were significantly associated with CAD susceptibility under different genetic models in the current sample of Egyptian CAD patients. These variants interacted with different traditional risk factors to cluster the current study population into the patient and control subgroups by multivariate analysis application. However, there are some limitations in our study should be considered. First, the study is cross-sectional for the point of the coronary disease investigation and all the study participants were stable CAD patients. Thus, the clinical phenotype was determined up to the time that the study was performed thereby allowing us to detect an association, but not to predict the outcome. Hence, follow-up studies are highly recommended to estimate the effect of the study risk factors on the disease outcome. In addition, including CAD patients with different clinical presentations are recommended to be useful to differentiate the distribution of the genetic pattern analyzed. Second, it is possible that some of our control subjects could have subclinical atherosclerosis that might result in an underestimation of the true risk associated with certain alleles. Additional studies examining coronary angiography free controls, will be of even greater value to more clearly establish the magnitude of risk imparted by the presence of these genetic risk factors. Third, the lack of an association between some variants and CAD may be due to the relatively small sample size that warrant a larger cohort in the same population and in different ethnicity populations to validate the current results.

## Additional files

Additional file 1: Table S1. Structural characteristics and functional impact of the studied single nucleotide polymorphisms. **Table S2** Odds ratio (95% confidence intervals) for the association between genotypes of thrombotic gene polymorphisms and CAD risk within strata of cardiovascular risk factors. **Table S3** Odds ratio (95% confidence intervals) for the association between genotypes of atherosclerotic gene polymorphisms and CAD risk within strata of cardiovascular risk factors. **Table S4** Correlation analysis matrix between genotypes and cardiovascular risk factors. (DOCX 41 kb)
Additional file 2: Figure S1. Genotyping results of the study CAD patients. (DOCX 174 kb)

## Abbreviations

ACE: Angiotensin-converting enzyme
APC: Activated protein C
Apo B: Apolipoprotein B
BMI: Body mass index
CAD: Coronary artery disease
CVD: Cardiovascular disease
Cx: Circumflex coronary artery
DCA: Detrended correspondence analysis
FBG: Fasting blood glucose
FGB: Beta-fibrinogen
FV (L): Factor V Leiden
FXIII: Factor 13
HDL: High density lipoprotein
HPA-1: Human platelet antigen 1
LAD: Left anterior descending
LDL: Low density lipoprotein
LMCA: Left main coronary artery
MTHFR: 5,10-Methylenetetrahydrofolate Reductase
MVD: Multivessel disease
PAI-1: Plasminogen activator inhibitor 1
PTH: Prothrombin
RCA: Right coronary artery
SDS: Sodium dodecyl sulfate
TC: Total cholesterol
TG: Triglycerides
