# Supplementary material for: Atherosclerotic and thrombotic genetic and environmental determinants in Egyptian coronary artery disease patients: a pilot study
Source: BMC Cardiovasc Disord. 2017 Jan 13;17:26. doi: 10.1186/s12872-016-0456-3 (PMC5237236; doi:10.1186/s12872-016-0456-3)
Supplement: Additional file 1: Table S1. — Structural characteristics and functional impact of the studied single nucleotide polymorphisms. Table S2 Odds ratio (95% confidence intervals) for the association between genotypes of thrombotic gene polymorphisms and CAD risk within strata of cardiovascular risk factors. Table S3 Odds ratio (95% confidence intervals) for the association between genotypes of atherosclerotic gene polymorphisms and CAD risk within strata of cardiovascular risk factors. Table S4 Correlation analysis matrix between genotypes and cardiovascular risk factors. (DOCX 41 kb) [file 12872_2016_456_MOESM1_ESM.docx]

**Additional file 1**

**Table S1 Structural characteristics and functional impact of the studied single nucleotide polymorphisms**

| **Gene (SNP ID)** | **Genomic location (strand)** | **Allele change** | **Residue change** | **Other synonym** | **MAF** | **Function** | **PolyPhen annotation (score)** | **SIFT prediction (score)** |
| --- | --- | --- | --- | --- | --- | --- | --- | --- |
| *F5: Leiden* (rs6025) | 1: 169549811 (minus) | c.1691G>A  CGA/CAA | R534Q | c.1601G>A | 0.01 (T) | Missense (exon 10) | Probably damaging (0.984) | Deleterious (0.0) |
| *F5: R2* (rs1800595) | 1: 169541110 (minus) | 4070A>G  CAT/CGT | H1299R | H1327R  c.4125A>G | 0.04 (C) | Missense (exon 13) | Benign (0.038) | Tolerated (0.05) |
| *F2* (rs1799963) | 11: 46739505 (plus) | 20210G>A | NA | c.*97G>A | < 0.01 (A) | 3' UTR variant | NA | NA |
| *F13A1* (rs5985) | [6:6318562](http://www.ensembl.org/Homo_sapiens/Location/View?contigviewbottom=variation_feature_variation%3Dnormal;db=core;r=6:6318512-6318612;tl=7Pb9cHGZYpYsiuDT-1696985) (minus) | c.103G>T  GTG/TTG | V34L | V89L | 0.22 (T) | Missense (exon 2) | Benign (0.0) | Tolerated (1.0) |
| *MTHFR* (rs1801133) | 1: 11796321 (minus) | 667C>T  GCC/GTC | A222V | 665C>T | 0.25 (A) | Missense (exon 5) | Probably damaging (0.996) | Deleterious (0.02) |
| *MTHFR* (rs1801131) | 1: 11794419 (plus) | 1298A>C  GAA/GCA | E470A | E429A | 0.25 (G) | Missense | Benign (0.005) | Tolerated (0.15) |
| *PAI-1* (rs1799889) | 7:101126430 (plus) | -675 4G/5G | NA |  |  | Del/ins  (promoter) | NA | NA |
| *FGB* (rs1800790) | 4: [154562556](http://www.ncbi.nlm.nih.gov/variation/view/?q=rs1800790&filters=source:dbsnp&assm=GCF_000001405.28) (plus) | -455G>A | NA | c.-463G>A | 0.141 (A) | Upstream gene variant | NA | NA |
| HPA-1 (rs5918) | 17:  47283364 (plus) | c.176T>C (a/b)  CTG/CCG | L59P |  | 0.09 (C) | Missense (exon 3) | Benign (0.002) | Tolerated (0.28) |
| *ACE* (rs1799752) | 17: 63488529-63488530 (plus) | I/D | NA |  | 2.591e-05 (G) | Intronic variant (Intron 16) | NA | NA |
| *ApoB* (rs5742904) | 2: 21006288 (minus) | c.10580G>A  CGG/CAG | R3500Q | p.Arg3527Gln | <0.001 (A) | Missense (exon 26) | Probably damaging (0.968) | Deleterious (0.0) |
| *ApoE* (rs429358 and rs7412) | 19: 44919684  +  19: 44908822 | c.388T>C  TGC/CGC c.526C>T  CGC/TGC | C130R  R176C | E2/E3/E4 | 0.15 (C)  0.08 (T) | Missense (exon 4) | Probably damaging (1.0) | Deleterious (0.0) |

SNP, Single nucleotide polymorphism; F5, coagulation factor V; F2, Prothrombin; F13A1, coagulation factor 13 alpha 1 chain; *MTHFR*, Methylenetetrahydrofolate reductase; *FGB*, fibrinogen beta polypeptide chain; *PAI-1*, plasminogen activator inhibitor 1 (SERPINE1); *HPA-1*, human platelet antigen 1, *ACE*, angiotensin converting enzyme; *ApoB*, Apolipoprotein B-100; *ApoE*, apolipotprotein E; MAF, Minor allele frequency; SIFT, sorts intolerant from tolerant amino acid substitutions [Data source: SNPedia and ensemble.org according to the Human assembly GRCh38.p2, Annotation release 107].

**Table S2 Odds ratio (95 % confidence intervals) for the association between genotypes of thrombotic gene polymorphisms and CAD risk within strata of cardiovascular risk factors.**

| **Potential confounder** | **Strata** | **Association** | ***F5: Leiden* (rs6025)** | ***F5: R2* (rs1800595 )** | ***F13A1* (rs5985)** | ***MTHFR* (rs1801133)** | ***MTHFR* (rs1801131)** | ***PAI-1* (rs1799889)** |
| --- | --- | --- | --- | --- | --- | --- | --- | --- |
| **Gender** | Female | P value | 0.065 | 0.100 | 1.000 | 0.149 | **0.026** | 0.400 |
|  |  | OR (95% CI) | 1.5 (0.85-2.64) | 7.0 (0.79-61.9) | 1.4 (0.1-19.01) | 0.12 (0.01-1.28) | 0.16 (0.007-3.8)  4.0 (0.21-75.6) ^a^  0.7 (0.05-9.7) ^b^ | 0.33 (0.035-3.2)  0.5 (0.18-1.3) ^a^  0.2 (0.03-2.7) ^b^ |
|  | Male | P value | 1.00 | 0.121 | 0.228 | **0.007** | 0.395 | 0.587 |
|  |  | OR (95% CI) | 0.5 (0.04-6.08) | 4.36 (0.74-25.7) | 3.3 (0.55-20..2) | 5.7 (0.88-36.8)  **24.2 (1.2-496)^a^**  **11.4 (1.9-66.5)^b^** | 2.0 (0.4-9.83)  0.57 (0.10-3.18) ^a^  1.1 (0.29-4.3) ^b^ | 1.6 (0.3-7.8)  2.4 (0.44-12.9) ^b^  1.9 (0.47-7.7) ^b^ |
| **Obesity** | Negative | P value | 0.365 | **0.038** | 0.09 | **0.013 ^#^** | 0.276 | 0.88 |
|  |  | OR (95% CI) | 2.6 (0.38-17.3) | **4.8 (1.17-19.6)** | 50 (0.85-29.3) | 1.1 (0.26-4.6)  **23.7 (1.2-474)^a^**  2.5 (0.72-8.5) ^b^ | 0.3 (0.06-1.36)  0.64 (0.14-2.93) ^a^  0.42 (0.11-1.58) ^b^ | 1.4 (0.33-6.0)  1.33 (0.2-7.6) ^a^  1.4 (0.35-5.5) ^b^ |
|  | Positive | P value | NA | 0.455 | 1.00 | 0.567 | 0..231 | **0.046** |
|  |  | OR (95% CI) | NA | 1.25 (0.80-193) | 0.5 (0.03-7.99) | 0.33 (0.02-3.9) | 6.0 (0.35-101)  2.0 (0.5-7.99) ^a^  80 (0.5-127) ^b^ | 0.33 (0.1-1.03)  1.6 (0.8-3.4) ^a^  0.33 (0.02-3.9) ^b^ |
| **FH** | Negative | P value | 0.636 | 0.071 | 0.465 | 0.104 | 0.390 | 0.291 |
|  |  | OR (95% CI) | 2.1 (0.31-14.54) | 4.4 (0.93-20.9) | 1.8 (0.41-8.43) | 0.66 (0.16-2.6)  9.2 (0.4-200) ^a^  1.06 (0.3-3.7) ^b^ | 0.6 (0.12-2.79)  1.8 (0.31-10.2) ^a^  0.9 (0.22-3.66) ^b^ | 0.35 (0.07-1.6)  1.07 (0.19-5.9) ^a^  0.5 (0.13-2.05) ^b^ |
|  | Positive | P value | NA | 0.245 | 0.333 | 0.099 | 0.710 | 0.256 |
|  |  | OR (95% CI) | NA | 5.5 (.38-78.5) | 1.2 (.83-1.71) | 1.6 (0.11-25.4)  15.0 (0.6-394) ^a^  5.0 (0.55-45.3) ^b^ | 0.66 (0.06-6.41)  0.33 (0.02-4.73) ^a^  0.5 (00.06-3.69) ^b^ | **0.6 (0.36-0.9)**  0.33 (0.1-1.03) ^a^  **0.66 (0.4-0.9)** **^b^** |
| **DM** | Negative | P value | 0.632 | **0.008** | 0.632 | **0.020** | 0.793 | 0.841 |
|  |  | OR (95% CI) | 2.3 (0.33-15.7) | **10.0 (1.7-57.7)** | 2.3 (0.33-15.7) | 2.6 (0.6-11.5)  **22.8 (1.07-487)** **^a^**  **4.4 (1.1-17.6)** **^b^** | 0.6 (0.13-2.73)  0.66 (0.12-3.6) ^a^  .6 (0.15-2.4) ^b^ | 1.2 (0.2-5.4)  1.6 (0.3-9.2) ^a^  1.3 (0.3-5.17) ^b^ |
|  | Positive | P value | NA | 1.00 | 0.305 | **0.047** | 0.435 | 0.298 |
|  |  | OR (95% CI) | NA | 0.83 (0.06-11.2) | 3.7 (0.44-31.6) | 0.07 (0.003-1.6)  3.0 (0.10-88.1) ^a^  016 (0.01-1.8) ^b^ | 0.66 (0.06-6.87)  3.0 (0.25-35.3) ^a^  1.2 (0.16-9.5) ^b^ | 1.25 (0.8-1.9)  1.5 (0.67-3.3) ^a^  1.16 (0.86-1.5) ^b^ |

*F5*, coagulation factor V; *F13A1*, coagulation factor 13 alpha 1 chain; *MTHFR*, Methylenetetrahydrofolate reductase; *PAI-1*, plasminogen activator inhibitor 1; FH; family history of cardiovascular disease, DM; diabetes mellitus, NA; not applicable. No history of smoking, hypertension and dyslipidemia among controls, thus excluded from the traditional risk factors. F2 (rs1799963) is not included as all participants were normal. Fisher's exact and two-sided Chi-square tests were used to compare between patients and controls in each strata. *P* values <0.05 are statistically significant. OR (95 %); odds ratio (95 % confidence interval) under heterozygote comparison (heterozygote versus wild homozygote), homozygote comparison (mutant versus wild homozygote) ^a^ and dominant model (heterozygote and mutant versus wild homozygote) ^b^.

**Table S3 Odds ratio (95% confidence intervals) for the association between genotypes of atherosclerotic gene polymorphisms and CAD risk within strata of cardiovascular risk factors.**

| **Potential confounder** | **Strata** | **Association** | ***FGB* (rs1800790)** | ***HPA-1* (rs5918)** | ***ACE* (rs1799752)** | ***ApoE* (**[**rs429358**](http://snpedia.com/index.php/Rs429358) **and** [**rs7412**](http://snpedia.com/index.php/Rs7412)**)** |
| --- | --- | --- | --- | --- | --- | --- |
| Gender | Female | P value | 0.783 | 0.065 | **<0.001** | **0.017** |
|  |  | OR (95% CI) | 0.5 (0.06-3.9)  1.0 (0.06-15.9)  0.6 (0.09-3.9) ^b^ | 1.5 (0.85-2.6) | 0.87 (0.72-1.05)  0.87 (0.72-1.05) ^b^ | 0.85 (069-1.06) ^c^  **30.0 (2.19-410) ^d^** |
|  | Male | P value | 0.057 | 0.315 | 0.121 | **0.032** |
|  |  | OR (95% CI) | **6.2 (1.05-36.5)**  1.1 (0.9-1.36)  **7.1 (1.2-40.9) ^b^** | 0.83 (0.21-3.34)  1.2 (0.92-1.6) ^a^  1.11 (0.29-4.2) ^b^ | 1.33 (0.19-9.0)  7.0 (0.69-70.7) ^a^  2.14 (0.33-13.5) ^b^ | 1.75 (0.92-3.32) ^c^  3.12 (0.70-13.8) ^d^ |
| Obesity | Negative | P value | 0.416 | 0.193 | **<0.001** | 0.156 |
|  |  | OR (95% CI) | 2.2 (0.62-8.1)  2.25 (0.26-18.9) ^b^ | 1.13 (0.29-4.3)  1.1 (0.93-1.4) ^a^  1.5 (0.45-5.5) ^b^ | 1.27 (0.12-13.5)  20.0 (1.39-287) ^a^  2.8 (0.29-27.6) ^b^ | 3.2 (0.35-28.9) ^c^  3.52 (0.94-13.1) ^d^ |
|  | Positive | P value | 1.000 | 0.061 | 0.231 | **0.004** |
|  |  | OR (95% CI) | 0.5 (0.03-7.9) | 2.5 (0.85-7.3) | 1.0 (0.05-18.9)  3.0 (0.60-14.8) ^a^  2.0 (0.12-31.9) ^b^ | NA |
| FH | Negative | P value | 0.865 | 0.081 | **0.011** | 0.110 |
|  |  | OR (95% CI) | 1.45 (0.29-7.09)  1.45 (0.17-11.9) ^b^ | 2.9 (0.71-12.3)  1.25 (0.91-1.7) ^a^  3.8 (0.96-15.1) ^b^ | **1.0 (0.15-6.64)**  **11.2 (1.1-106)** **^a^**  2.2 (0.37-13.1) ^b^ | 4.0 (0.38-41.2) ^c^  **6.0 (1.27-28.25) ^d^** |
|  | Positive | P value | 0.316 | 1.000 | **0.025** | **0.013** |
|  |  | OR (95% CI | 5.0 (0.44-56.6 | 0.6 (0.04-7.40 | 0.91 (0.77-1.08) | 1.5 (0.67-3.33) ^c^  7.5 (0.71-78.3) ^d^ |
| DM | Negative | P value | 0.105 | 0.092 | **0.006** | **0.040** |
|  |  | OR (95% CI) | 6.0 (1.003-35.9)  1.0 (0.08-12.5) ^b^ | 2.6 (0.65-10.8)  1.2 (0.9-1.82) ^a^  3.4 (0.8-13.3) ^b^ | 1.5 (0.13-16.2)  **18.0 (1.24-260) ^a^**  3.33 (0.33-33.1) ^b^ | 1.45 (0.85-2.64) ^c^  **4.3 (1.02-18.6) ^d^** |
|  | Positive | P value | 0.324 | 1.00 | **0.045** | **0.046** |
|  |  | OR (95% CI) | 0.5 (0.06-3.6)  1.3 (0.75-2.3) ^b^ | 0.83 (0.06-11.2) | 0.6 (0.03-9.15)  4.0 (0.73-21.8) ^a^  1.2 (0.08-16.2) ^b^ | 4.0 (0.16-95.7) ^c^  **20.0 (1.41-282.4) ^d^** |

*FGB*, fibrinogen beta polypeptide chain; *HPA-1*, human platelet antigen 1, *ACE*, angiotensin converting enzyme; FH; family history of cardiovascular disease, *ApoE*, apolipotprotein E; DM; diabetes mellitus, NA; not applicable. No history of smoking, hypertension and dyslipidemia among controls, thus excluded from the traditional risk factors. *ApoB* (rs5742904) is not included as all participants were normal. Fisher's exact and two-sided Chi-square tests were used. *P* values < 0.05 are statistically significant (i.e.bold data). OR (95 %); odds ratio (95% confidence interval) under heterozygote comparison (heterozygote versus wild homozygote), homozygote comparison (mutant versus wild homozygote) ^a^ and dominant model (heterozygote and mutant versus wild homozygote) ^b^. For *ApoE* SNP: OR (95 % CI) for E3/4 versus E2 ^c^ and E3/E3 versus E2 ^d^.

| **Table S4 Correlation analysis matrix between genotypes and cardiovascular risk factors** | | | | | | | | | | |
| --- | --- | --- | --- | --- | --- | --- | --- | --- | --- | --- |
|  | ***FVL*** | ***FVR*** | ***F13A1*** | ***MTHFR1*** | ***MTHFR2*** | ***PAI-1*** | ***FGB*** | ***HPA-1*** | ***ACE*** | ***ApoE*** |
| **Age** | -0.185 (0.168) | 0.053 (0.693) | -0.014 (0.917) | -0.167 (0.215) | 0.104 (0.440) | 0.145 (0.282) | 0.104 (0.440) | **-0.352 (0.007)** | -0.248 (0.063) | -0.039 (0.774) |
| **Gender** | -0.009 (0.947) | 0.002 (0.991) | 0.081 (0.547) | -0.083 (0.539) | -0.204 (0.129) | -0.012 (0.930) | **-0.319 (0.016)** | **0.386 (0.003)** | 0.034 (0.801) | 0.126 (0.350) |
| **BMI** | -0.230 (0.085) | -0.131 (0.331) | 0.253 (0.058) | 0.208 (0.121) | -0.091 (0.500) | 0.070 (0.602) | 0.129 (0.340) | 0.117 (0.388) | -0.1989 (0.140) | -0.198 (0.140) |
| **Obesity** | -0.152 (0.260) | -0.160 (0.235) | 0.125 (0.354) | 0.092 (0.498) | -0.199 (0.137) | -0.132 (0.327) | -0.147 (0.277) | -0.055 (0.685) | -0.151 (0.262) | -0.076 (0.573) |
| **Smoking** | -0.169 (0.210) | 0.103 (0.445) | 0.298 (0.023) | 0.120 (0.375) | -0.073 (0.588) | 0.055 (0.683) | 0.139 (0.301) | 0.207 (0.123) | 0.047 (0.729) | 0.248 (0.063) |
| **FH** | -0.211 (0.116) | -0.099 (0.462) | -0.214 (0.110) | -0.142 (0.290) | -0.055 (0.684) | 0.037 (0.782) | 0.224 (0.094) | -0.147 (0.275) | -0.005 (0.969) | **-0.273 (0.039)** |
| **HTN** | 0.183 (0.173) | 0.189 (0.159) | 0.030 (0.826) | 0.039 (0.775) | 0.027 (0.842) | 0.061 (0.652) | 0.217 (0.105) | -0.026 (0.848) | **0.378 (0.003)** | 0.091 (0.500) |
| **DM** | -0.219 (0.101) | -0.118 (0.381) | 0.163 (0.225) | 0.065 (0.631) | 0.000 (1.000) | **0.258 (0.052)** | **0.289 (0.028)** | -0.247 (0.064) | -0.125 (0.353) | 0.027 (0.842) |
| **Dyslipidemia** | 0.175 (0.192) | 0.237 (0.076) | **0.261 (0.049)** | 0.258 (0.053) | 0.098 (0.469) | 0.031 (0.820) | 0.151 (0.261) | **0.340 (0.010)** | **0.315 (0.017)** | **0.295 (0.026)** |
| **FBS** | 0.098 (0.468) | 0.140 (0.299) | 0.036 (0.788) | -0.116 (0.389) | -0.172 (0.200) | -0.154 (0.252) | 0.036 (0.789) | 0.097 (0.472) | 0.197 (0.143) | **0.267 (0.044)** |
| **TC** | -0.106 (0.434) | 0.022 (0.873) | 0.032 (0.812) | **0.371 (0.004)** | -0.220 (0.100) | 0.003 (0.985) | 0.139 (0.301) | 0.083 (0.540) | 0.086 (0.526) | 0.098 (0.466) |
| **TG** | 0.025 (0.856) | **0.368 (0.004)** | 0.231 (0.083) | 0.087 (0.520) | 0.141 (0.297) | 0.063 (0.640) | -0.145 (0.280) | 0.162 (0.230) | **0.282 (0.033)** | **0.398 (0.002)** |
| **HDL** | -0.164 (0.222) | **-0.288 (0.030)** | 0.030 (0.828) | -0.030 (0.827) | -0.182 (0.176) | -0.603 (0.641) | -0.027 (0.843) | -0.226 (0.091) | **-0.302 (0.022)** | -0.066 (0.628) |
| **LDL** | -0.015 (0.911) | 0.219 (0.102) | **0.264 (0.047)** | 0.253 (0.057) | **0.2668 (0.044)** | 0.122 (0.364) | **0.267 (0.043)** | **0.298 (0.024)** | 0.259 (0.052) | **0.314 (0.017)** |

Data are shown as correlation coefficient (*p* values). Spearman's rank correlation coefficient was used. A two-tailed *p* value of < 0.05 was considered statistically significant (i.e.bold data). *FVL*, coagulation factor 5 Leiden; *F13A1*, coagulation factor 13 alpha 1 chain; *MTHFR*, Methylenetetrahydrofolate reductase; *PAI-1*, plasminogen activator inhibitor 1; *FGB*, fibrinogen beta polypeptide chain; *HPA-1*, human platelet antigen 1, *ACE*, angiotensin converting enzyme; *ApoE*, apolipotprotein E; BMI, body mass index; FH; family history of cardiovascular disease, HTN, hypertension; DM; diabetes mellitus, FBS, fasting blood sugar; TC, total cholesterol; TG, triglycerides; HDL, high density lipoprotein; LDL; low density lipoprotein.
