# Supplementary material for: Atherosclerotic and thrombotic genetic and environmental determinants in Egyptian coronary artery disease patients: a pilot study
Source: BMC Cardiovasc Disord. 2017 Jan 13;17:26. doi: 10.1186/s12872-016-0456-3 (PMC5237236; doi:10.1186/s12872-016-0456-3)
Supplement: Additional file 2: Figure S1. — Genotyping results of the study CAD patients. (DOCX 174 kb) [file 12872_2016_456_MOESM2_ESM.docx]

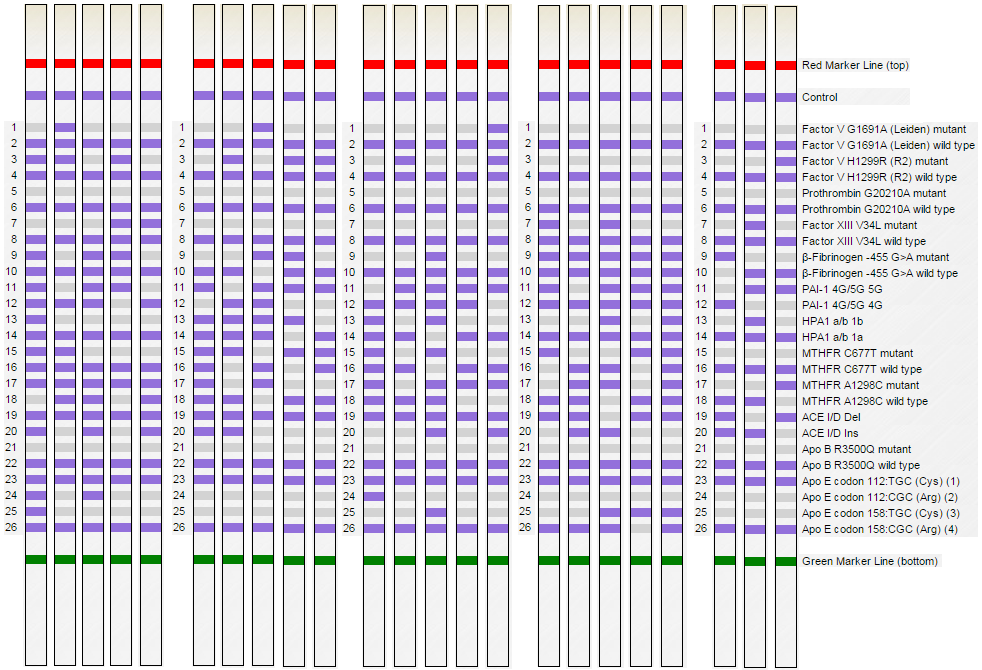


**Additional file 2:Figure S1 Genotyping results of the study CAD patients (n = 23).** Case No. 1 (from left) to case No. 23 (right). Signals pattern of the study strip bands were translated into schematic results using stripassay® online calculator (http://viennalab.sci-design.at/webevaluator/).
